# Antimicrobial Susceptibility of *Vibrio vulnificus* and *Vibrio parahaemolyticus* Recovered from Recreational and Commercial Areas of Chesapeake Bay and Maryland Coastal Bays

**DOI:** 10.1371/journal.pone.0089616

**Published:** 2014-02-25

**Authors:** Kristi S. Shaw, Rachel E. Rosenberg Goldstein, Xin He, John M. Jacobs, Byron C. Crump, Amy R. Sapkota

**Affiliations:** 1 Horn Point Laboratory, University of Maryland, Center for Environmental Science, Cambridge, Maryland, United States of America; 2 Maryland Institute for Applied Environmental Health, University of Maryland, School of Public Health, College Park, Maryland, United States of America; 3 Department of Epidemiology and Biostatistics, University of Maryland, School of Public Health, College Park, Maryland, United States of America; 4 Cooperative Oxford Laboratory, National Oceanic and Atmospheric Administration, National Ocean Service, National Centers for Coastal Ocean Science, Oxford, Maryland, United States of America; 5 College of Earth, Ocean, and Atmospheric Sciences, Oregon State University, Corvallis, Oregon, United States of America; Rockefeller University, United States of America

## Abstract

*Vibrio vulnificus* and *V. parahaemolyticus* in the estuarine-marine environment are of human health significance and may be increasing in pathogenicity and abundance. *Vibrio* illness originating from dermal contact with *Vibrio* laden waters or through ingestion of seafood originating from such waters can cause deleterious health effects, particularly if the strains involved are resistant to clinically important antibiotics. The purpose of this study was to evaluate antimicrobial susceptibility among these pathogens. Surface-water samples were collected from three sites of recreational and commercial importance from July to September 2009. Samples were plated onto species-specific media and resulting *V. vulnificus* and *V. parahaemolyticus* strains were confirmed using polymerase chain reaction assays and tested for antimicrobial susceptibility using the Sensititre® microbroth dilution system. Descriptive statistics, Friedman two-way Analysis of Variance (ANOVA) and Kruskal-Wallis one-way ANOVA were used to analyze the data. *Vibrio vulnificus* (n = 120) and *V. parahaemolyticus* (n = 77) were isolated from all sampling sites. Most isolates were susceptible to antibiotics recommended for treating *Vibrio* infections, although the majority of isolates expressed intermediate resistance to chloramphenicol (78% of *V. vulnificus*, 96% of *V. parahaemolyticus*). *Vibrio parahaemolyticus* also demonstrated resistance to penicillin (68%). Sampling location or month did not significantly impact *V. parahaemolyticus* resistance patterns, but *V. vulnificus* isolates from St. Martin's River had lower overall intermediate resistance than that of the other two sampling sites during the month of July (*p* = 0.0166). Antibiotics recommended to treat adult *Vibrio* infections were effective in suppressing bacterial growth, while some antibiotics recommended for pediatric treatment were not effective against some of the recovered isolates. To our knowledge, these are the first antimicrobial susceptibility data of *V. vulnificus* and *V. parahaemolyticus* recovered from the Chesapeake Bay. These data can serve as a baseline against which future studies can be compared to evaluate whether susceptibilities change over time.

## Introduction

Bacterial antimicrobial resistance is a critical public health issue of increasing importance for those who recreate and work in coastal regions. Pathogenic bacteria and antimicrobial resistance genes are often released with wastewater discharges into aquatic environments [1]. Naturally occurring bacteria produce antibiotics in the environment for signaling and regulatory purposes in microbial communities [2]. Bacteria protect themselves from the toxicity of these antibiotics by acquiring and expressing antibiotic resistance genes [3]. As a result, naturally-occurring aquatic bacteria are capable of serving as reservoirs of resistance genes and those genes, coupled with the introduction and accumulation of antimicrobial agents, detergents, disinfectants, and residues from industrial processes, may play an important role in the evolution and spread of antibiotic resistance in aquatic environments [1].

*Vibrio* bacteria in the estuarine-marine environment are of particular concern for human health and may be increasing in pathogenicity and abundance [4]. Cases of vibriosis are rising in the United States, with *Vibrio vulnificus* and *V. parahaemolyticus* being two of the three most commonly reported sources of *Vibrio* infection [5]. *V. parahaemolyticus* is implicated as the primary source of escalation in vibriosis incidence [5] and highly pathogenic serotypes of this species are emerging on a global scale, including the Atlantic coasts of the United States and Spain [6]. It is estimated that only 1 in 142 cases of *V. parahaemolyticus* illness is detected [7]. Calculations based upon probable incidence of vibriosis have estimated that *V. vulnificus* and *V. parahaemolyticus* are the first and third most costly marine-borne pathogens, costing $233 and $20 million, respectively[8].

Antimicrobial susceptibility patterns among *Vibrio* spp. inhabiting estuarine-marine environments may have implications for recreational and commercial users of these environments, and for those who consume *Vibrio*-contaminated seafood. Previous studies exploring antimicrobial susceptibility of *Vibrio vulnificus* and *V. parahaemolyticus* have been conducted in South Carolina, the United States Gulf region and Italy [9], [10], [11], [12]. However, to our knowledge, no similar studies have been completed in the Chesapeake Bay, the largest estuary in the U.S., which lies in a watershed where 17 million people work, live and play.

The work of our group and others has demonstrated that concentrations of *V. vulnificus* and *V. parahaemolyticus* in the Chesapeake Bay are high enough to result in possible illnesses among exposed recreationists, particularly among those who are immunocompromised [13], [14], [15], [16], [17], [18]. Moreover, current models predict that total tissue loading of shellfish and finfish with *V. vulnificus* and *V. parahaemolyticus* is associated not only with surface water concentrations but also with the risk of illness for those consuming contaminated seafood products [19], [20], [21]. Given these data, along with the knowledge that environmental conditions may be increasingly more favorable for *Vibrio* growth [22], it is not surprising that rates of *Vibrio* infections are increasing in Maryland and other U.S. states [23]. In this context, it is critical to gain a better understanding of the antimicrobial susceptibility patterns of *V. vulnificus* and *V. parahaemolyticus* originating from estuarine-marine environments.

This study evaluated antimicrobial susceptibility patterns of *V. vulnificus* and *V. parahaemolyticus* recovered from the Chesapeake Bay and Maryland Coastal Bays. Our findings provide the first antimicrobial susceptibility data among *Vibrio* bacteria isolated from this region. These data will be helpful in short and long-term predictions of human health risks associated with exposures to *Vibrio* populations in the Chesapeake Bay area.

## Materials and Methods

### Sampling sites

**Figure 1:**
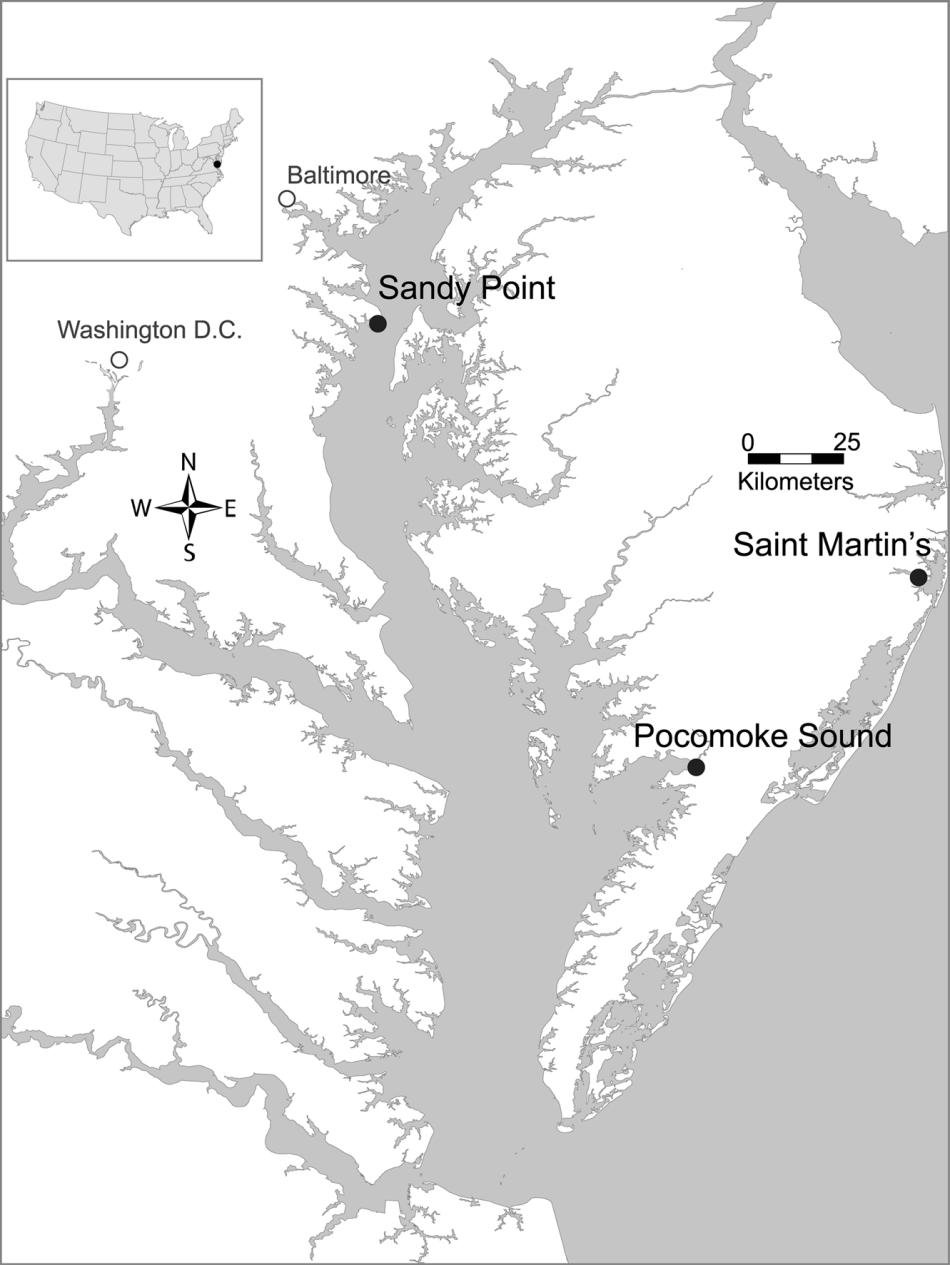
Sampling sites in Chesapeake Bay and Maryland Coastal Bays. (Tracey Saxby, Kate Boicourt, Integration and Application Network, University of Maryland Center for Environmental Science (ian.umces.edu/imagelibrary/displayimage-127-5815.html).

Three sampling sites were selected based on their importance for human use in the Chesapeake Bay, Maryland Coastal Bays region. Two sites, Sandy Point State Park and St. Martin's River, were characterized by frequent recreational use; and one site, the Pocomoke Sound, was characterized by heavy commercial fishing use (Figure 1). Sandy Point State Park includes an artificial beach on the western shore of the Chesapeake mid-Bay region, at the base of the Chesapeake Bay Bridge. It is open year round and frequented by approximately 768,000 visitors annually, many of whom visit the park's beach during the summer (Sandy Point Park staff, Maryland Department of Natural Resources, personal communication). St. Martin's River is a tributary of the Maryland Coastal Bays with approximately 10,000 residents. Land-use in the St. Martin's River watershed is approximately 10% residential, 48% agricultural, and 34% forested [24]. The Pocomoke Sound is a major embayment of the Chesapeake Bay's Eastern Shore. It is influenced by agricultural practices, including high-density concentrated poultry feeding operations, and is a popular destination for commercial and recreational fishing. No specific permissions were required for each sampling location, as they are public access waterways, and no endangered or protected species were involved in sampling activities.

### Sample collection

Sampling dates were chosen to coincide with times of high recreational and/or commercial use. Surface water samples (n = 9) were collected during Summer 2009, once a month, at each site, for three consecutive months (July, August, September) within two hours of high tide and on approximately the same date each month. Water samples were collected just below the surface in sterile wide mouth polypropylene 1 L environmental sampling bottles (Nalgene Thermo Scientific, Waltham, MA). Bottles were rinsed three times with surface water and then dipped below the surface for a final 1 L collection volume. Samples collected for *Vibrio* culture were kept in insulated coolers, while water samples for enterococci culture were stored in an insulated container on ice (4°C) upon collection, returned to the laboratory within four hours and processed immediately upon arrival.

### Physical and chemical water quality measurements

Water-column depth and surface-water salinity, temperature, dissolved oxygen, conductivity, and pH were measured on every sampling date and at each location with a YSI 556 Multi-probe system (YSI Incorporated, Yellow Springs, OH) in accordance with the manufacturer's instructions.

### Fecal indicator measurements

Fecal indicator measurements were conducted following the standard methods as described for enterococci in Standard Methods for the Examination of Water and Wastewater [25]. Briefly, surface-water samples were filtered in triplicate onto sterile 0.45 µm pore size, 47 mm diameter, nitrocellulose Fisherbrand water-testing membrane filters (Fisher Scientific, Pittsburgh, PA), and plated onto Difco m *Enterococcus* (BD, Franklin Lakes, NJ) agar. According to manufacturer's instructions, plates were incubated for 48 hours at 35°C. All light to dark red colonies were recorded as presumptive enterococci.

### *Vibrio* isolation

Surface water samples (100 µL) were spread plated in triplicate onto Chromagar *Vibrio* media (DRG International, Mountainside, NJ) and incubated for 24 hours at 37°C. After incubation, each plate was observed for characteristically colored bacterial colonies associated with *V. vulnificus* (turquoise) or *V. parahaemolyticus* (mauve). As *V. vulnificus* and *V. cholerae* both appear as turquoise colonies on Chromagar *Vibrio* media, all turquoise colonies were replated onto cellobiose-collistin (CC) agar (FDA 2004) media to confirm *V. vulnificus* species. The CC agar cultures were incubated for 24 hours at 37°C and yellow-colored colonies were considered presumptive *V. vulnificus.* Tryptic soy broth (TSB), supplemented with 5% sodium chloride, was then inoculated with individual presumptive colonies of *V. vulnificus* or *V. parahaemolyticus* and incubated at 37°C for 24 hours and stored with 30% glycerol at −80°C.

### *Vibrio* species confirmation

*Vibrio* DNA template was obtained by producing crude cell lysates by boiling 1 mL aliquots of TSB cultures in 2 mL micro-centrifuge tubes at 100°C for 10 minutes. A Bio-rad CFX96 Touch Real-Time PCR Detection System (Bio-rad, Hercules, CA, USA) was used to confirm the species of isolates with primers designed to detect *Vibrio vulnificus* [26] or *V. parahaemolyticus* [27]. Following initial confirmation, samples testing positive for either species were subjected to further testing for virulence genes (*V. vulnificus*: virulence correlated gene clinical variant (*vcgC*) [28]; *V. parahaemolyticus*: thermostable direct hemolysin (*tdh*), and thermostable related hemolysin (*trh*) genes [27]) using real-time PCR.

Real-time PCR was performed by using 1X PCR Buffer (Qiagen, Valencia, CA), (Qiagen), 0.2 mM dNTP's solution (Qiagen), 1X Q solution (Qiagen), 2.25U TopTaq DNA polymerase (Qiagen), 75 nM internal control primers (each), 150 nM internal control probe, 2 µL internal control DNA, target primer and probe concentrations as detailed in Table 1, and 3 µL DNA template per reaction, with the exception of the Vv *vcgC* assay, where 5 µL of DNA template was used and the internal control components were absent. DNase-RNase free water was added in a quantity sufficient for a 25 uL total reaction volume. Two-stage qPCR cycling parameters are presented in Table 1. A linear synthetic exogenous DNA internal control, including a primer set, probe and internal control DNA, was incorporated simultaneously into each assay (excluding the assays for the *V. vulnificus vcgC* target) to test for the presence and influence of inhibitors (Nordstrom et al., 2007). The following positive controls were used in each qPCR: *Vibrio parahaemolyticus* USFDA TX2103 and *Vibrio vulnificus* ATCC 27562.

**Table 1:** PCR conditions for the detection of *V. vulnificus* and *V. parahaemolyticus* virulence genes.

	Primer (forward & reverse)/	
Primer	Probe Concentrations (nM)	PCR conditions
*Vibrio vulnificus*/*vvh*	400/240	1x: 95°C for 60 s; 41x: 95°C for 5 s, 59°C for 45 s
*Vibrio vulnificus*/*vcgC*	250/180	1x: 95°C for 10 m; 40x: 95°C for 15 s, 60°C for 90 s
*Vibrio parahaemolyticus*/*tlh*	200/150	1x: 95°C for 10 m; 45x: 95°C for 5 s, 66°C for 45 s
*Vibrio parahaemolyticus*/*tdh trh*	200/75	1x: 95°C for 60 s; 50x: 95°C for 5 s, 59°C for 45 s

A randomly chosen subset of isolates were taxonomically identified with 16 S rRNA gene sequences. DNA extracted from cultures was PCR-amplified with bacteria-specific primers 27f (5′-AGAGTTTGATCCTGGCTCAG-3′) and 907r (5′-CCGTCAATTCCTTTRAGTTT-3′) using the following conditions: 94°C for 2 min, followed by 25 cycles of 55°C for 30 s, 72°C for 30 s, and 94°C for 2 min, followed by 72°C for 5 min. The PCR products were sequenced bi-directionally using the same primers on an ABI 3730 XL Genetic Analyzer in the BioAnalytical Services Laboratory at the University of Maryland Center for Environmental Science. Paired reads for each organism were analyzed and assembled with Phred and Phrap [29], [30], manually edited with Consed [31], and aligned and analyzed with the ARB sequence alignment program [32]. DNA sequences were deposited in the GenBank database under accession numbers KF990336 to KF990363.

### Clinical isolates

Clinical isolates of *V. parahaemolyticus* (n = 8) were graciously provided by the State of Maryland's Department of Health and Mental Hygiene for comparison purposes with our environmental isolates. Sample type and source of infection are presented in Table 2.

**Table 2:** Sample type, infection source, and antimicrobial resistance of clinical *V. parahaemolyticus* isolates provided by the Maryland Department of Health and Mental Hygiene.

Clinical	Sample		Antibiotic Resistance	
isolate	type	Infection source	Intermediate resistance	Resistance
1	Stool	Undercooked seafood	Ampicillin, Penicillin	None
2	Stool	Undercooked seafood	Chloramphenicol	Ampicillin, Penicillin
3	Stool	No data available	Chloramphenicol, Penicillin	None
4	Stool	Undercooked seafood	Chloramphenicol, Apramycin, Streptomycin	Ampicillin, Penicillin
5	Stool	Undercooked seafood	Chloramphenicol	None
6	Stool	No data available	Chloramphenicol, Ampicillin	Penicillin
7	No data available	Beach, unknown location	Chloramphenicol, Ampicillin, Penicillin	None
8	Wound	No data available	Chloramphenicol	Ampicillin, Penicillin

### Antimicrobial susceptibility testing

Antimicrobial susceptibility testing was performed using the Sensititre® microbroth dilution system (Trek Diagnostic Systems, Westlake, Ohio) in accordance with the manufacturer's instructions on all PCR-confirmed *V. vulnificus* (n = 120 (3 *vcgC*+)) and *V. parahaemolyticus* (n = 77 (1 *tdh*+, 1 *trh*+)). Cultures were grown overnight on tryptic soy agar (TSA)+2.5% NaCl plates at 37°C. *Vibrio* cultures were transferred to sterile demineralized 2.5% saline solution to achieve a 0.5 McFarland standard. Then, 100 µL of each suspension was transferred to sterile cation-adjusted Mueller Hinton broth (Trek Diagnostic Systems, Westlake, Ohio), and 50 µL of the broth solution was dispensed into CML1FMAR custom minimal inhibitory concentration (MIC) plates (Trek Diagnostic Systems Inc.) with the following 26 antibiotics (range of concentrations in μg/ml): amikacin (AMI; 8-64), ampicillin (AMP; 4-32), ampicillin-sulbactam 2∶1 (A/S2; 8/4-32/16), apramycin (APR; 8-32), cefoxitin (FOX; 8-32), ceftriaxone (AXO; 8-64), cephalothin (CEP; 8-128), chloramphenicol (CHL; 8-32), ciprofloxacin (CIP; 1-4), oflaxacin (OFL; 1-8), ceftazidime (TAZ; 8-32), cefepime (FEP; 8-32), cefotaxime (FOT; 8-64), meropenem (MERO; 2-16), doxycycline (DOX; 2-16), imipenem (IMI; 2-16), levofloxacin (LEVO; 2-8), cefuroxime (FUR; 8-32), trimethoprim-sulfamethoxazole (SXT; 2/38-4/76), penicillin (PEN; 16-128), piperacillin (PIP; 16-128); piperacillin-tazobactam (P/T4; 16/4-128/4), streptomycin (STR; 8-128), tetracycline (TET; 4-32), gentamicin (GEN; 2-16), and amox/clav 2∶1(AUG2; 8/4-32/16). *Escherichia coli* ATCC 25922 and *E.coli* ATCC 35218 were used as quality control strains. MICs were recorded as the lowest concentration of an antimicrobial that completely inhibited bacterial growth [33]. Resistance breakpoints published by the Clinical and Laboratory Standards Institute were used [33]. Breakpoints not available from CLSI (streptomycin, apramycin, penicillin) were derived from ranges used in similar studies [9], [10], [34], [35]. Multidrug resistance (MDR) was defined as resistance to two or more antibiotics.

### Statistical analyses

Descriptive statistics were used to compare the percentage of isolates demonstrating intermediate resistance or resistance to tested antibiotics at each sampling site and sampled month, as well as the average number of antibiotics that *V. vulnificus* and *V. parahaemolyticus* isolates were resistant to at each sampling location and during each month. Nonparametric Friedman two-way Analysis of Variance (ANOVA) was used to determine effects related to sampling site and month sampled. For samples for which month influenced percent resistance, stratified Kruskal-Wallis one-way ANOVA and pairwise post-hoc tests were conducted for each month separately to evaluate differences in the occurrence of antimicrobial susceptibility between strains that carried or did not carry virulence genes. All statistical analyses were performed using StataIC 12 and *p*-values of ≤0.05 were defined as statistically significant. (StatCorp LP, College Station, TX).

## Results

### Physical, chemical and bacterial water quality

Water temperature, pH, and dissolved oxygen (DO) were uniform across the three sampling locations (Table 3). Average salinity (± standard deviation) in St. Martin's River (24.5 ppt (±1.07)) was approximately double that of the Pocomoke Sound (10.5 ppt (±0.54)) and Sandy Point State Park (9.4 ppt (±0.72)) sampling sites. Water depth at the Pocomoke Sound was approximately double that of Sandy Point State Park and three to four-fold that of St. Martin's River.

**Table 3:** Physical, chemical and bacterial water quality including salinity (S), temperature (t), dissolved oxygen (DO), depth (d), and Average concentration of *Enterococcus* (geometric mean CFU 100 mL^−1^), *V. vulnificus (Vv)*, and *V. parahaemolyticus (Vp)* (CFU mL^−1^).

Site	Date	S	T	pH	DO	d	E*nterococcus*	*Vv*	*Vp*
			(°C)		(mg L^−1^)	(m)			
Pocomoke	16-Jul-2009	10.5	26.1	7.6	n/a	4.8	24 (8)	51 (41)	13 (9)
Pocomoke	18-Aug-2009	10	28.8	7.4	4.9	4.4	15 (10)	35 (29)	8 (9)
Pocomoke	21-Sep-2009	11.1	22.6	7.3	6.3	4.2	38 (6)	52 (40)	9 (10)
Sandy Point	9-Jul-2009	8.6	24.5	8.3	7.4	2.3	2 (3)	204 (137)	11 (23)
Sandy Point	3-Aug-2009	10	26.5	8	7	2.3	5 (4)	234 (76)	19 (15)
Sandy Point	3-Sep-2009	9.6	24.6	7.8	7.1	2.3	2 (3)	294 (71)	18 (11)
St. Martin's	6-Jul-2009	24.5	25.9	7.9	6.6	1.3	3 (7)	28 (46)	17 (20)
St. Martin's	9-Aug-2009	23.4	26.5	7.8	5.6	1.5	365 (6)	122 (47)	48 (40)
St. Martin's	6-Sep-2009	25.5	23.1	7.5	2.9	1.5	3 (5)	32 (24)	12 (12)

Standard deviations are in parentheses.

Enterococci counts (colony forming units (CFU)) per 100 ml^−1^ were uniformly low at Sandy Point during each sampling time point and below the single sample regulatory closure level of 10^4^ CFU per 100 ml^−1^ [36]. On one sampling occasion in St. Martin's River (August) enterococci counts exceeded closure levels (Table 3).

Presumptive *Vibrio* colonies isolated during this study indicated that *V. vulnificus* and *V. parahaemolyticus* were present in all tested water samples (Table 3). One-hundred twenty *V. vulnificus* and 77 *V. parahaemolyticus* isolates were purified, confirmed via PCR and tested for antimicrobial susceptibility.

### *Vibrio* species and virulence identification

**Figure 2:**
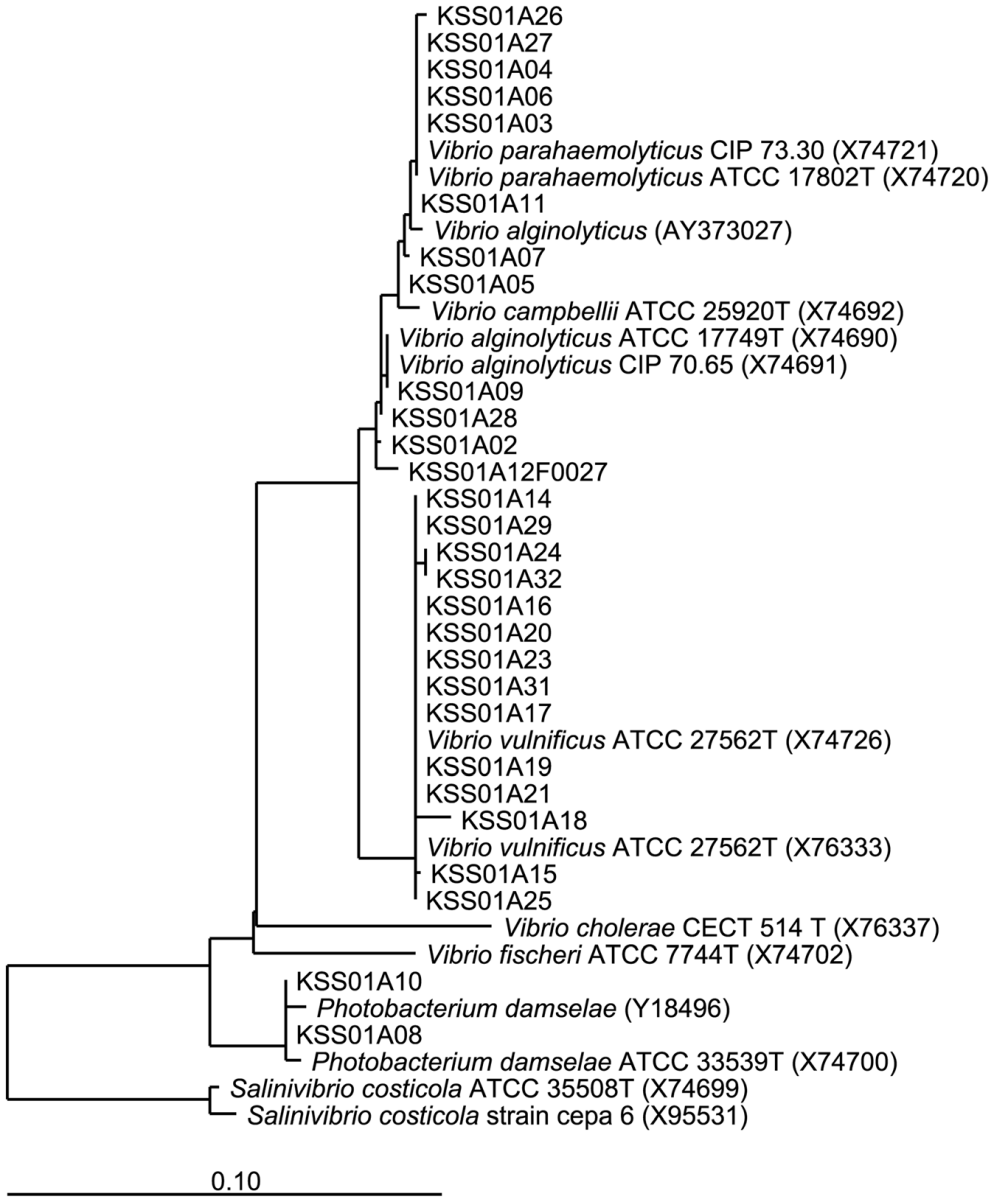
16S rRNA sequencing analysis of a subset of *Vibrio* isolates tested.

Sequence analysis (16S rRNA) of a selected subset of tested *Vibrio* isolates confirmed all isolates (Figure 2), except for two isolates with sequences similar to *Photobacterium damselae*. Virulence testing of all isolates identified three *V. vulnificus* isolates positive for *vcgC*, one *V. parahaemolyticus* isolate positive for *tdh*, and one *V. parahaemolyticus* isolate positive for *trh*.

### Prevalence of antimicrobial resistance in *V. vulnificus*

**Figure 3:**
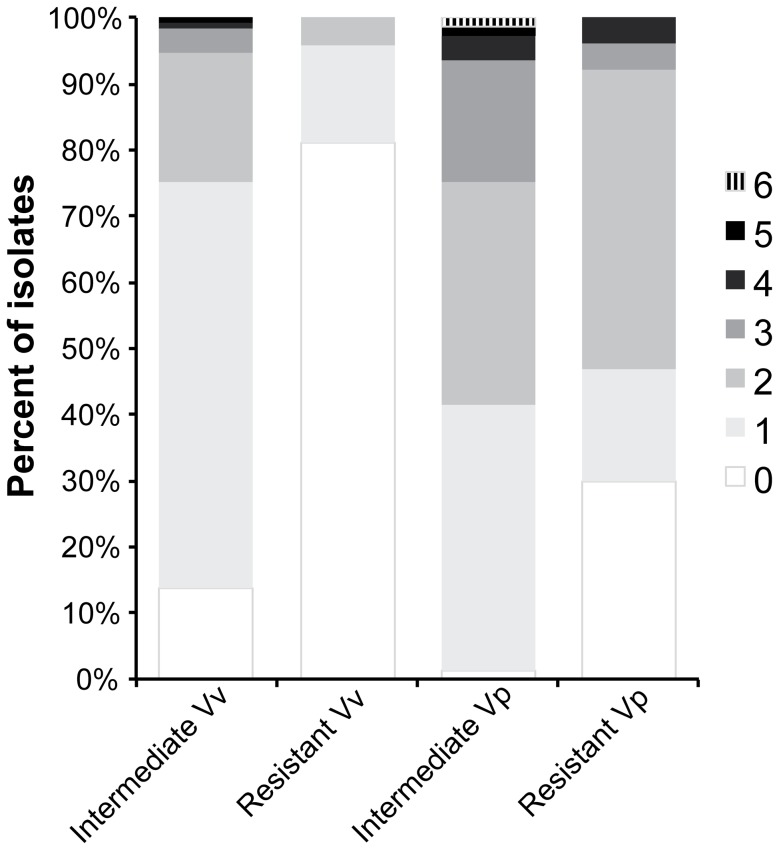
Number of antibiotics against which *Vibrio vulnificus (Vv) and Vibrio parahaemolyticus (Vp)* isolates expressed resistance or intermediate resistance.

All tested *V. vulnificus* isolates (n = 120) were susceptible to 14 of the 26 antibiotics tested, including the following drug classes that are recommended by the Centers for Disease Control and Prevention (CDC) for the treatment of *V. vulnificus* infections: tetracyclines, quinolones, and folate pathway inhibitors (Table 4, Figure 3). With regard to CDC recommended antimicrobial agents, 2% of the tested isolates exhibited intermediate resistance against ceftazidime, a third generation cephalosporin. Within the aminoglycoside class of antibiotics, isolates exhibited resistance to apramycin (1%) and streptomycin (4%). Intermediate resistance was expressed against amikacin (1%), apramycin (5%) and streptomycin (8%). Gentamicin was the only tested aminoglycoside to which all *V. vulnificus* isolates were completely susceptible. The aminoglycoside streptomycin was associated with the highest percentage of resistance (7% of all tested isolates) and second highest percentage of intermediate resistance (17% of all tested isolates) out of all of the antimicrobials tested. Isolates displayed the highest percentage of intermediate resistance (78% of all isolates) to chloramphenicol.

**Table 4:** Antimicrobial resistance patterns among environmental *Vibrio* isolates.

	Penicillins and β-Lactam/β-Lactamase Inhibitor Combinations						Cephems							Carba-penems		Aminoglycosides				Tetra-cyclines		Quinolones			Other	
	Ampicillin (≥32)	Amoxicillin-clavulanic acid (≥32/16)	Ampicillin-sulbactam (≥32/64)	Penicillin (≥64)	Piperacillin (≥128)	Piperacillin-tazobactam (≥128/4)	Cefepime (≥32)	Cefotaxime** (≥64)	Cefoxitin (≥32)	Ceftazidime** (≥32)	Ceftriaxone** (≥64)	Cefuroxime sodium (≥32)	Cephalothin (≥32)	Imipenem (≥16)	Meropenem (≥16)	Amikacin** (≥64)	Apramycin** (≥64)	Gentamicin** (≥16)	Streptomycin** (≥64)	Doxycycline** (≥16)	Tetracycline** (≥16)	Ciprofloxacin** (≥4)	Levofloxacin** (≥8)	Oflaxacin (≥8)	Chloramphenicol (≥32)	Trimethoprim-sulfamethoxazole** (≥4/76)
*V. vulnificus* (n = 120)																										
# Susceptible	115	118	120	116	119	118	120	120	108	118	119	120	114	118	120	117	106	120	90	120	119	120	120	120	26	120
# Intermediate	3	0	0	0	1	0	0	0	6	2	0	0	4	0	0	3	9	0	20	0	0	0	0	0	94	0
# Resistant	1	0	0	4	0	1	0	0	5	0	0	0	2	1	0	0	5	0	8	0	0	0	0	0	0	0
% Susceptible	97	100	100	97	99	99	100	100	91	98	100	100	95	99	100	98	88	100	76	100	100	100	100	100	22	100
% Intermediate	3	0	0	0	1	0	0	0	5	2	0	0	3	0	0	3	8	0	17	0	0	0	0	0	78	0
% Resistant	1	0	0	3	0	1	0	0	4	0	0	0	2	1	0	0	4	0	7	0	0	0	0	0	0	0
*V. parahaemolyticus* (n = 77)																										
# Susceptible	17	76	76	13	71	76	73	75	74	77	77	65	63	77	77	76	72	76	68	77	77	77	77	77	3	77
# Intermediate	19	1	0	12	1	0	1	2	3	0	0	11	13	0	0	1	4	0	6	0	0	0	0	0	74	0
# Resistant	40	0	1	52	3	1	1	0	0	0	0	1	1	0	0	0	1	0	3	0	0	0	0	0	0	0
% Susceptible	22	99	99	17	95	99	97	97	96	100	100	84	82	100	100	99	94	100	88	100	100	100	100	100	4	100
% Intermediate	25	1	0	16	1	0	1	3	4	0	0	14	17	0	0	1	5	0	8	0	0	0	0	0	96	0
% Resistant	53	0	1	68	4	1	1	0	0	0	0	1	1	0	0	0	1	0	4	0	0	0	0	0	0	0

**CDC recommended.

### Antimicrobial resistance in vcgC+ *V. vulnificus*

Of the three isolates positive for the virulence correlated gene clinical variant (*vcgC*), none displayed resistance to any of the tested antibiotics, but all three expressed intermediate resistance (100%) to chloramphenicol.

### Prevalence of antimicrobial resistance in *V. parahaemolyticus*

All tested *V. parahaemolyticus* isolates were susceptible to 11 of the 26 tested antibiotics and four (carbapenems, tetracyclines, quinolones and folate pathway inhibitors) of the eight tested antimicrobial classes (Table 4). Conversely, 96% of isolates were characterized by intermediate resistance to chloramphenicol, followed by ampicillin (25%), cephalothin (17%), penicillin (16%) and cefuroxime sodium (14%). A high percentage of resistance was observed against some of the penicillins (penicillin (68%); ampicillin (53%)), while a low percentage of resistance was seen against piperacillin (4%) and streptomycin (4%).

### Antimicrobial resistance in *tdh*/*trh*+ *V. parahaemolyticus*

One *V. parahaemolyticus* isolate was *tdh*+ and one isolate was *trh*+. The *trh*+ *V. parahaemolyticus* isolate was resistant to ampicillin and penicillin and expressed intermediate resistance to chloramphenicol. The *tdh*+ *V. parahaemolyticus* isolate was resistant to ampicillin, ampicillin-sulbactam, penicillin, piperacillin-tazobactam, and amoxicillin-clavulanic acid and expressed intermediate resistance to chloramphenicol.

### Impact of sampling site and month on antimicrobial resistance

#### Friedman two-way ANOVA:

The month when sampling occurred significantly influenced rates of antibiotic resistance and intermediate resistance among *V. parahaemolyticus* (*p*<0.0001, *p*<0.0001, respectively), as well as resistance and intermediate resistance among *V. vulnificus* (*p* = 0.0008, *p* = 0.0098, respectively). After adjusting for the repeated measures over time (month), sampling site also significantly influenced resistance and intermediate resistance among *V. vulnificus* (*p* = 0.0321, *p* = 0.0029, respectively), but not among *V. parahaemolyticus* (*p* = 0.6133, *p* = 0.7660, respectively).

#### Kruskal-Wallis one-way ANOVA:

As there was a significant month effect in the Friedman two-way ANOVA for both *V. vulnificus* and *V. parahaemolyticus* isolates expressing antibiotic resistance and intermediate resistance, stratified Kruskal-Wallis one-way ANOVA and pairwise post-hoc tests were conducted on the sampling site differences for each month separately. Results showed no significant difference between sampling sites by month for *V. vulnificus* or *V. parahaemolyticus* expressing resistance (July, August, September; (*p* = 0.5340, 0.2801, 0.4966); (*p* = 0.7246, 0.9448, 0.6809), respectively) or *V. parahaemolyticus* expressing intermediate resistance (*p* = 0.5959, 0.8046, 0.2135). After testing *V. vulnificus* expressing intermediate resistance for sampling site differences by each month separately, it was determined that there was a significant sampling site effect only in July (*p* = 0.035). Post-hoc testing clarified that the site, St. Martin's River, was different from Sandy Point during the month of July, with reduced intermediate resistance among *V. vulnificus* isolates recovered from St. Martin's River (*p* = 0.0166).

Kruskal-Wallis one-way ANOVA further elucidated that there was no significant difference in the median intermediate resistance or resistance patterns during the sampling period when St. Martin's River (August) (*p* = 0.44) had higher levels of bacterial-indicator species.

### Clinical V. parahaemolyticus

Clinical isolates tested displayed comparable resistance profiles to environmental isolates tested (Table 5). However, environmental isolates demonstrated intermediate resistance and resistance to a greater range of antibiotics (15 antibiotics in four classes) when compared to clinical isolates (five antibiotics in three classes). Yet, based on analyses with two-sample proportion tests, the overall percentage of resistance and intermediate resistance (% =  number of antimicrobials to which there was demonstrated resistance/number of total antimicrobials tested) among clinical isolates was not statistically different (*p* = 0.511, 0.430; respectively) from that of environmental isolates.

**Table 5:** Comparison of environmental and clinical *V. parahaemolyticus* isolates with regard to all antibiotics to which clinical isolates were found to have intermediate resistance or resistance.

	Environmental Isolates		Clinical Isolates	
Antibiotic	Intermediate n (%)	Resistant n (%)	Intermediate n (%)	Resistant n (%)
Ampicillin	19 (25)	40 (53)	1 (12.5)	2 (25)
Apramycin	4 (5)	1 (1)	1 (12.5)	0 (0)
Streptomycin	6 (8)	3 (4)	1 (12.5)	0 (0)
Chloramphenicol	74 (96)	0 (0)	7 (87.5)	0 (0)
Penicillin	12 (16)	52 (68)	3 (37.5)	4 (50)

Clinical isolates were susceptible to all other tested antibiotics.

Overall resistance profiles

The percentage of isolate resistance, defined as resistance to any one antibiotic (AR) or resistance to two or more classes of antibiotics is depicted in Table 6. Resistance profiles were comparable for isolates with and without detected virulence genes (6A), isolates from varying sampling locations (6B), and isolates recovered in different sampling months (6C) for both *V. parahaemolyticus* and *V. vulnificus*.

**Table 6:** Antibiotic resistance (AR), defined as resistance to any one antibiotic, and multi-drug resistance (MDR), defined as resistance to two or more antibiotic classes, by virulence factors (6A), site (6B) and month (6C) for *V. vulnificus* and *V. parahaemolyticus*.

			*V. vulnificus*			*V. parahaemolyticus*		
Category	grouping	Resistance	n	Res.	Int.	n	Res.	Int.
Virulence factors	Vv *vcgC*+	AR	3	0 (0%)	3 (100%)			
		MDR	3	0 (0%)	0 (0%)			
	Vv *vcgC-*	AR	117	21 (18%)	101 (86%)			
		MDR	117	0 (0%)	28 (24%)			
	Vp *tdh+*	AR				1	1 (100%)	1 (100%)
		MDR				1	0 (0%)	1 (100%)
	Vp *trh*+	AR				1	1 (100%)	1 (100%)
		MDR				1	0 (0%)	0 (0%)
	Vp *tdh*/*trh-*	AR				75	51 (68%)	74 (99%)
		MDR				75	4 (5%)	44 (59%)
Site	Pocomoke	AR	44	10 (23%)	42 (95%)	14	10 (71%)	14 (100%)
		MDR	44	0 (0%)	10 (23%)	14	1 (7%)	8 (57%)
	St. Martin's	AR	11	0 (0%)	6 (55%)	29	22 (76%)	28 (97%)
		MDR	11	0 (0%)	0 (0%)	29	2 (7%)	15 (52%)
	Sandy Point	AR	65	12 (18%)	58 (89%)	34	22 (65%)	34 (100%)
		MDR	65	0 (0%)	18 (28%)	34	1 (3%)	23 (68%)
Date	July	AR	40	3 (8%)	32 (80%)	11	9 (82%)	11 (100%)
		MDR	40	0 (0%)	4 (10%)	11	2 (18%)	7 (64%)
	August	AR	47	13 (28%)	42 (89%)	40	31 (78%)	39 (98%)
		MDR	47	0 (0%)	15 (32%)	40	0 (55%)	24 (60%)
	September	AR	33	6 (18%)	30 (91%)	26	14 (54%)	26 (100%)
		MDR	33	0 (0%)	9 (27%)	26	2 (7%)	14 (54%)

## Discussion

### Treatability of Chesapeake Bay related *Vibrio* illness

*Vibrio vulnificus* and *V. parahaemolyticus* are the causative agents for wound infections, primary septicemia, and gastroenteritis related to seafood and seawater exposure [37]. While antibiotic treatment is not typically necessary for gastroenteritis, it is required for wound infection and primary septicemia caused by both *Vibrio* species analyzed in this study. Most isolates tested in this study were susceptible to the antimicrobial agents recommended by the CDC for clinical treatment. Treatment recommendations for *Vibrio* infections include tetracyclines (doxycycline, tetracycline), flouroquinolones (ciprofloxacin, levofloxacin), third-generation cephalosporins (cefotaxime, ceftazidime, ceftriaxone), aminoglycosides (amikacin, apramycin, gentamicin, streptomycin) and folate pathway inhibitors (trimethoprim-sulfamethoxazole) [38], [39]. The CDC recommends a treatment course of doxycycline (100 mg PO/IV twice a day for 7-14 days) and a third-generation cephalosporin (e.g.,ceftazidime 1–2 g IV/IM every eight hours), although they state that single agent regimens employing a fluoroquinolone have been reported to be at least as effective in an animal model as combination drug regimens with doxycycline and a cephalosporin [39].

All tested *V. vulnificus* isolates were susceptible to third and fourth generation cephalosporins, although two *V. parahaemolyticus* isolates (3%) demonstrated intermediate resistance to cefotaxime, a third-generation cephalosporin, and two isolates demonstrated a degree of resistance to cefepime, a fourth-generation cephalosporin. While the percentage of isolates expressing intermediate resistance and resistance to the newer generation cephalosporins was relatively low, these antibiotics are considered to be some of the best defenses against the severe infections that these organisms can elicit, so even a small percentage of resistant isolates could be cause for concern [39].

Due to the contraindication of doxycycline and fluoroquinolones in children, a combination of trimethoprim-sulfamethoxazole and an aminoglycoside antibiotic is recommended [39]. Given that three of the four tested aminoglycosides (amikacin, apramycin, streptomycin) were associated with intermediate resistance or resistance (e.g., streptomycin intermediate resistance and resistance in *V. vulnificus*: 17%, 7%, respectively; *V. parahaemolyticus*: 8%, 4%, respectively) in a subset of isolates, this may be a resistance pattern of concern. Conversely, for the aminoglycoside, gentamicin, all tested isolates were fully susceptible. Based on these data, physicians in the Bay region may consider focusing on gentamicin as the aminoglycoside of choice in multi-drug treatment regimens for *Vibrio* infections contracted by children recreating in the Chesapeake Bay.

### Comparison to other studies of *V. vulnificus* and *V. parahaemolyticus* antimicrobial susceptibility

The percent resistance among *Vibrios*, in this study was comparable to a similar study conducted on *Vibrios* isolated from Gulf Coast oysters in Louisiana [11]. Han et al. (2007) also found higher levels of resistance among *V. parahaemolyticus* compared to *V. vulnificus* isolates. In addition, ampicillin was the only tested antimicrobial in the Gulf Coast study to which a large percentage of *V. parahaemolyticus* isolates demonstrated intermediate resistance to resistance (∼81% of all tested isolates). This trend was seen as early as the 1970s in a study that tested resistance of *V. parahaemolyticus* to ampicillin and β-lactamase inhibitors [40], where over 90% of isolates were found to be resistant to ampicillin. In contrast to the present study, Han et al. (2007) found no resistance in either *Vibrio* species to chloramphenicol, cefotaxime, or ceftazidime, while we observed intermediate resistance against these three antimicrobial agents among a subset of *V. vulnificus* (78%, 0%, 2%, respectively) and *V. parahaemolyticus* (96%, 3%, 0%, respectively).

Our findings are also in partial agreement with two large studies of *V. vulnificus* and *V. parahaemolyticus* isolates originating from the Georgia and South Carolina coastline of the United States [10]. While our Chesapeake Bay isolates did not show the same high prevalence of antimicrobial resistance, the antimicrobial agents to which isolates displayed resistance were similar (i.e., amoxicillin, apramycin, penicillin and streptomycin for *V. parahaemolyticus*). *V. vulnificus* isolates demonstrated similar resistance profiles, particularly with regard to percent intermediate resistance and resistance to the penicillin class and cefoxitin. Baker-Austin et al. (2009) reported higher percent intermediate resistance and resistance among *V. vulnificus* against apramycin and streptomycin compared to that of the isolates reported in our study. In addition, key antimicrobials to which *V. parahaemolyticus* isolates from Georgia/South Carolina displayed susceptibility were also found to be susceptibile in our study (i.e., ceftriaxone, ciprofloxacin, imipenem, ofloxacin, meropenem, tetracycline), except in the case of chloramphenicol, for which no or low (*V. vulnificus*) resistance was observed in the Georgia and South Carolina study. In contrast to this study, Baker-Austin et al. (2009) found only one (<0.01%) *V. vulnificus* isolate to be completely susceptible to all antimicrobials tested, while the present study found 15 (12.5%) isolates to be susceptible to all tested antimicrobials.

A recent study of antimicrobial susceptibility in toxigenic and non-toxigenic *V. parahaemolyticus* isolates from shellfish and clinical samples in Italy [12] produced interesting comparisons to our findings. Similar to other studies, no intermediate resistance or resistance to chloramphenicol was found in Italian *V. parahaemolyticus* samples, whereas our study found high levels of intermediate resistance to this antibiotic. The Italian study found isolates to be 100% (n = 170) resistant to ampicillin, while our study detected 53% (n = 40) resistance and 25% (n = 25) intermediate resistance. Resistance to cefotaxime was found in approximately 20% (n = 21) of Italian samples, compared to 0% resistance and 4% (n = 3) intermediate resistance in this study. In contrast to our study, which detected full susceptibility to ciprofloxacin, the Italian study found resistance (9%, n = 10), particularly in clinical samples. Comparable susceptibility patterns are reported in these studies for trimethoprim-sulfamethoxazole, doxycycline and tetracycline, as all *V. parahaemolyticus* tested in this study were fully susceptible to these three antibiotics, while Italian isolates displayed intermediate resistance for trimethoprim-sulfamethoxazole (4%, n = 4) and tetracycline (11%, n = 12).

### Sampling sites and influences of pollution

Each sampling site included in this study has a history of water pollution. Sandy Point State Park has historically been a site of low bacteriological water quality and is adjacent to the Magothy River, a site where there have been numerous wastewater treatment overflows. The Pocomoke River is located adjacent to many agricultural operations, including poultry concentrated animal feeding operations (CAFOs), which may increase the introduction of antimicrobial residues into the waterway due to runoff of fecal matter contaminated with antimicrobials used in poultry production [41]. Finally, St. Martin's River is adjacent to many homes on septic systems, notorious for leakage [42]. While each of the sampling sites has a history of contamination that may increase the incidence of antimicrobial residues and associated changes in resident bacteria in the estuarine environment, this study only detected a small difference in levels of antibiotic resistance between sites. Specifically, in the month of July, *V. vulnificus* recovered from St. Martin's River expressed higher percentages of intermediate resistance compared to isolates recovered from Sandy Point.

However, it should be noted that this study was limited by the inability to culture *V. vulnificus* and *V. parahaemolyticus* from areas presumed to be void of contamination by human or animal sewage or industrial pollution. Due to this limitation, resistance levels detected at each of the three studied sites could not be compared to that of a local “pristine” site and this likely reduced our ability to differentiate pollution-related resistance from naturally occurring resistance among tested isolates.

### Antimicrobial susceptibility as compared to enterococci concentrations

In this study, we also tested for enterococci as an indicator of fecal contamination in order to specifically evaluate whether areas that were characterized by higher levels of possible fecal contamination were also marked by higher levels of antibiotic resistance. We observed a range of enterococci concentrations over the course of the study, although most sampling sites were within the range of acceptable water quality for recreation on each sampling date. Interestingly, concentrations of enterococci were not correlated with percentages of antibiotic resistance in the studied environments. During the one instance that the geometric mean of enterococci was higher than regulation limits, there was no discernible difference in levels of resistance among isolates originating from that site (St. Martin's River – August). This is counter to previous observations where percent antimicrobial resistance was elevated at sites contaminated with higher levels of enterococci that may have originated from fecal waste of humans [43] and animals [44].

## Conclusions

This study represents the first investigation of antimicrobial susceptibility of *Vibrio* species recovered from the Chesapeake Bay and provides a baseline against which future studies can be compared to determine whether susceptibilities change over time. Isolates tested in this study displayed high intermediate resistance to chloramphenicol, when compared to similar studies. Isolates' intermediate resistance and resistance to some aminoglycosides should be noted because these antibiotics are used to treat pediatric *Vibrio* illnesses originating from the Chesapeake waters or seafood. Low-level intermediate resistance and resistance to third and fourth generation cephalosporins may also limit treatment effectiveness and should be monitored. As most of the antimicrobial agents recommended for treatment of *Vibrio* illnesses by CDC were fully effective against *V. vulnificus* and *V. parahaemolyticus* isolated from the Chesapeake Bay, treating infections contracted from the Bay, at least in adults, is not likely to be problematic. Based on our data, treatment of pediatric illnesses may benefit from the use of trimethoprim-sulfamethoxazole and the aminoglycoside, gentamicin, which was the only aminoglycoside that was 100% effective against *Vibrios* recovered in this study.
